# Supplementary material for: Cocaine-induced gene regulation in D1 and D2 neuronal ensembles of the nucleus accumbens
Source: Commun Biol. 2025 Jun 12;8:919. doi: 10.1038/s42003-025-08327-x (PMC12163090; doi:10.1038/s42003-025-08327-x)
Supplement: Supplementary file 1 — Reporting Summary [file 42003_2025_8327_MOESM1_ESM.pdf]

Corresponding author(s): Philipp Mews, Eric J Nestler

Last updated by author(s): Feb 21, 2025

Reporting Summary

Nature Portfolio wishes to improve the reproducibility of the work that we publish. This form provides structure for consistency and transparency in reporting. For further information on Nature Portfolio policies, see our Editorial Policies and the Editorial Policy Checklist.

Statistics

For all statistical analyses, confirm that the following items are present in the figure legend, table legend, main text, or Methods section.

n/a	Confirmed	
☐	☒	The exact sample size (n) for each experimental group/condition, given as a discrete number and unit of measurement
☐	☒	A statement on whether measurements were taken from distinct samples or whether the same sample was measured repeatedly
☐	☒	The statistical test(s) used AND whether they are one- or two-sided<br>*Only common tests should be described solely by name; describe more complex techniques in the Methods section.*
☐	☒	A description of all covariates tested
☐	☒	A description of any assumptions or corrections, such as tests of normality and adjustment for multiple comparisons
☐	☒	A full description of the statistical parameters including central tendency (e.g. means) or other basic estimates (e.g. regression coefficient) AND variation (e.g. standard deviation) or associated estimates of uncertainty (e.g. confidence intervals)
☒	☐	For null hypothesis testing, the test statistic (e.g. F, t, r) with confidence intervals, effect sizes, degrees of freedom and P value noted<br>*Give P values as exact values whenever suitable.*
☒	☐	For Bayesian analysis, information on the choice of priors and Markov chain Monte Carlo settings
☒	☐	For hierarchical and complex designs, identification of the appropriate level for tests and full reporting of outcomes
☒	☐	Estimates of effect sizes (e.g. Cohen's d, Pearson's r), indicating how they were calculated

Our web collection on statistics for biologists contains articles on many of the points above.

Software and code

Policy information about availability of computer code

Data collection	The snRNAseq data were imported as distinct 10X Genomics files for each respective sample. Cell Ranger filtered outputs were analyzed with Seurat v4.4.0.
Data analysis	The snCell Ranger filtered outputs were analyzed with Seurat v4.4.0. Data normalization was performed using Seurat's LogNormalize. We employed an unsupervised clustering approach using the Seurat package to identify transcriptionally distinct subpopulations based on gene expression profiles without bias from treatment group assignments. The cell clustering pipeline was executed through Seurat, utilizing the Find Neighbors function with 17 dimensions, and determining clusters at a resolution of 0.25. The cluster genes were also analyzed through BioMart v2.56.1 for basic gene information. Upregulated genes specific to each cluster were further analyzed through Metascape 3.5 for GO term enrichment analysis and molecular complex detection. Marker gene lists were subjected to functional enrichment analysis using Metascape 3.5 online platform

For manuscripts utilizing custom algorithms or software that are central to the research but not yet described in published literature, software must be made available to editors and reviewers. We strongly encourage code deposition in a community repository (e.g. GitHub). See the Nature Portfolio guidelines for submitting code & software for further information.

Data

Policy information about availability of data

All manuscripts must include a data availability statement. This statement should provide the following information, where applicable:

- Accession codes, unique identifiers, or web links for publicly available datasets
- A description of any restrictions on data availability
- For clinical datasets or third party data, please ensure that the statement adheres to our policy

All data needed to evaluate the conclusions in the paper are present in the paper and/or the Supplementary Materials. All RNAseq data reported in this study will be deposited publicly in the Gene Expression Omnibus upon manuscript acceptance. Other supporting scripts/code used in this study are available from the corresponding author upon request.

Research involving human participants, their data, or biological material

Policy information about studies with human participants or human data. See also policy information about sex, gender (identity/presentation), and sexual orientation and race, ethnicity and racism.

Reporting on sex and gender	NA
Reporting on race, ethnicity, or other socially relevant groupings	NA
Population characteristics	NA
Recruitment	NA
Ethics oversight	NA

Note that full information on the approval of the study protocol must also be provided in the manuscript.

Field-specific reporting

Please select the one below that is the best fit for your research. If you are not sure, read the appropriate sections before making your selection.

☒ Life sciences ☐ Behavioural & social sciences ☐ Ecological, evolutionary & environmental sciences

For a reference copy of the document with all sections, see nature.com/documents/nr-reporting-summary-flat.pdf

Life sciences study design

All studies must disclose on these points even when the disclosure is negative.

Sample size	n = 2 for each D1 and D2 group. Male double-transgenic mice expressing D1- or D2-specific nuclear-tagged GFP were generated by crossing LSL-eGFP::L10a (IMSR_JAX:022367) mice with either D1-Cre (MGI:3836633) or D2-Cre (MGI:3836635) mice.
Data exclusions	No samples were excluded from the study.
Replication	Two biological replicates per each group for the snRNAseq.
Randomization	Animals were randomly assigned to groups.
Blinding	Blinded treatment of animals for tissue harvest and samples processing.

Reporting for specific materials, systems and methods

We require information from authors about some types of materials, experimental systems and methods used in many studies. Here, indicate whether each material, system or method listed is relevant to your study. If you are not sure if a list item applies to your research, read the appropriate section before selecting a response.

Materials & experimental systems

n/a	Involved in the study
☒	☐ Antibodies
☒	☐ Eukaryotic cell lines
☒	☐ Palaeontology and archaeology
☐	☒ Animals and other organisms
☒	☐ Clinical data
☒	☐ Dual use research of concern
☒	☐ Plants

Methods

n/a	Involved in the study
☒	☐ ChIP-seq
☒	☐ Flow cytometry
☒	☐ MRI-based neuroimaging

Animals and other research organisms

Policy information about studies involving animals; ARRIVE guidelines recommended for reporting animal research, and Sex and Gender in Research

Laboratory animals	Male double-transgenic mice expressing D1- or D2-specific nuclear-tagged GFP were generated by crossing LSL-eGFP::L10a (IMSR_JAX:022367) mice with either D1-Cre (MGI:3836633) or D2-Cre (MGI:3836635) mice. All mice were bred in-house on a C57BL/6J background and were 8-20 weeks old at the beginning of experimental procedures.
Wild animals	NA
Reporting on sex	Male C57BL/6J mice were used in this study.
Field-collected samples	NA
Ethics oversight	All experiments were conducted in accordance with the guidelines of the Institutional Animal Care and Use Committee (IACUC) at Mount Sinai (protocol number 08-0465).

Note that full information on the approval of the study protocol must also be provided in the manuscript.

Plants

Seed stocks	NA
Novel plant genotypes	NA
Authentication	NA
